# The active site residues Gln55 and Arg73 play a key role in DNA damage bypass by *S. cerevisiae* Pol η

**DOI:** 10.1038/s41598-018-28664-8

**Published:** 2018-07-09

**Authors:** Elizaveta O. Boldinova, Artem Ignatov, Andrey Kulbachinskiy, Alena V. Makarova

**Affiliations:** Institute of Molecular Genetics, Russian Academy of Sciences, Kurchatov sq. 2, 123182 Moscow, Russia

## Abstract

Eukaryotic DNA polymerase eta (Pol η) plays a key role in the efficient and accurate DNA translesion synthesis (TLS) opposite UV-induced thymine dimers. Pol η is also involved in bypass of many other DNA lesions but possesses low fidelity on undamaged DNA templates. To better understand the mechanism of DNA synthesis by Pol η we investigated substitutions of evolutionary conserved active site residues Gln55 and Arg73 in *Saccharomyces cerevisiae* Pol η. We analyzed the efficiency and fidelity of DNA synthesis by the mutant Pol η variants opposite thymine dimers, abasic site, thymine glycol, 8-oxoguanine and on undamaged DNA. Substitutions Q55A and R73A decreased the catalytic activity and significantly affected DNA damage bypass by Pol η. In particular, the Q55A substitution reduced the efficiency of thymine dimers bypass, R73A had a stronger effect on the TLS-activity opposite abasic site, while both substitutions impaired replication opposite thymine glycol. Importantly, the R73A substitution also increased the fidelity of Pol η. Altogether, these results reveal a key role of residues Gln55 and Arg73 in DNA synthesis opposite various types of DNA lesions and highlight the evolutionary importance of the Pol η TLS function at the cost of DNA replication accuracy.

## Introduction

Pol η is a eukaryotic Y-family DNA polymerase that participates in DNA translesion synthesis (TLS) and has been implicated in prevention of carcinogenesis induced by UV light. *S. cerevisiae* and human Pol η carry out efficient and accurate replication opposite *cis-syn* thymine dimers (cyclobutane pyrimidine dimers, CPD)^1,2^. Defects of the Pol η function in humans cause the genetic disease known as the variant form of xeroderma pigmentosum (XP-V phenotype) characterized by UV sensitivity and predisposition to skin cancer^3,4^. Pol η also bypasses 1,2-intrastrand d(GpG)-cisplatin adducts and contributes to the development of resistance to cisplatin chemotherapy in several human cancers, thus representing a promising target for the treatment of chemotherapy-resistant tumors^5–7^.

Pol η is also involved in DNA translesion synthesis (TLS) past various types of endogenous lesions, including oxidative DNA lesions^8–11^, abasic site (AP-site)^2,12–17^ and O^6^-methylguanine (O^6^-me-G)^18^. Human and yeast Pol η bypass 7,8-dihydro-8-oxoguanine (8-oxoG) *in vitro* and incorporate dCTP with slight preference over dATP opposite the lesion^8,11^. The role of Pol η in preventing of 8-oxo-G-induced mutagenesis *in vivo* was shown both in yeast and human cells^8,10^. Human Pol η also catalyzes efficient and accurate TLS opposite another oxidative lesion, thymine glycol (TG)^9^ and can efficiently bypass AP-sites *in vitro*, by incorporating dATP and dGTP opposite the lesion^2,14,15^. Yeast Pol η also incorporates dATP or dGTP opposite AP-site, depending on the sequence context^13^, but inefficiently extends the primer beyond the lesion^16^. The role of Pol η in the AP-site bypass in yeast *in vivo* remains uncertain^17,19,20^.

In contrast to replicative DNA polymerases, Pol η reveals very low fidelity when copying undamaged DNA with misincorporation rates of 10^−2^–10^−3^ ^21–23^. Like all Y-family DNA polymerases, Pol η lacks the proofreading 3′–5′ exonuclease activity^22^. The error-prone replication by Pol η contributes to the A/T mutagenesis at the WA motif (TA or AA) during somatic hypermutation of immunoglobulin genes in mammals^24–26^.

**Figure 1:**
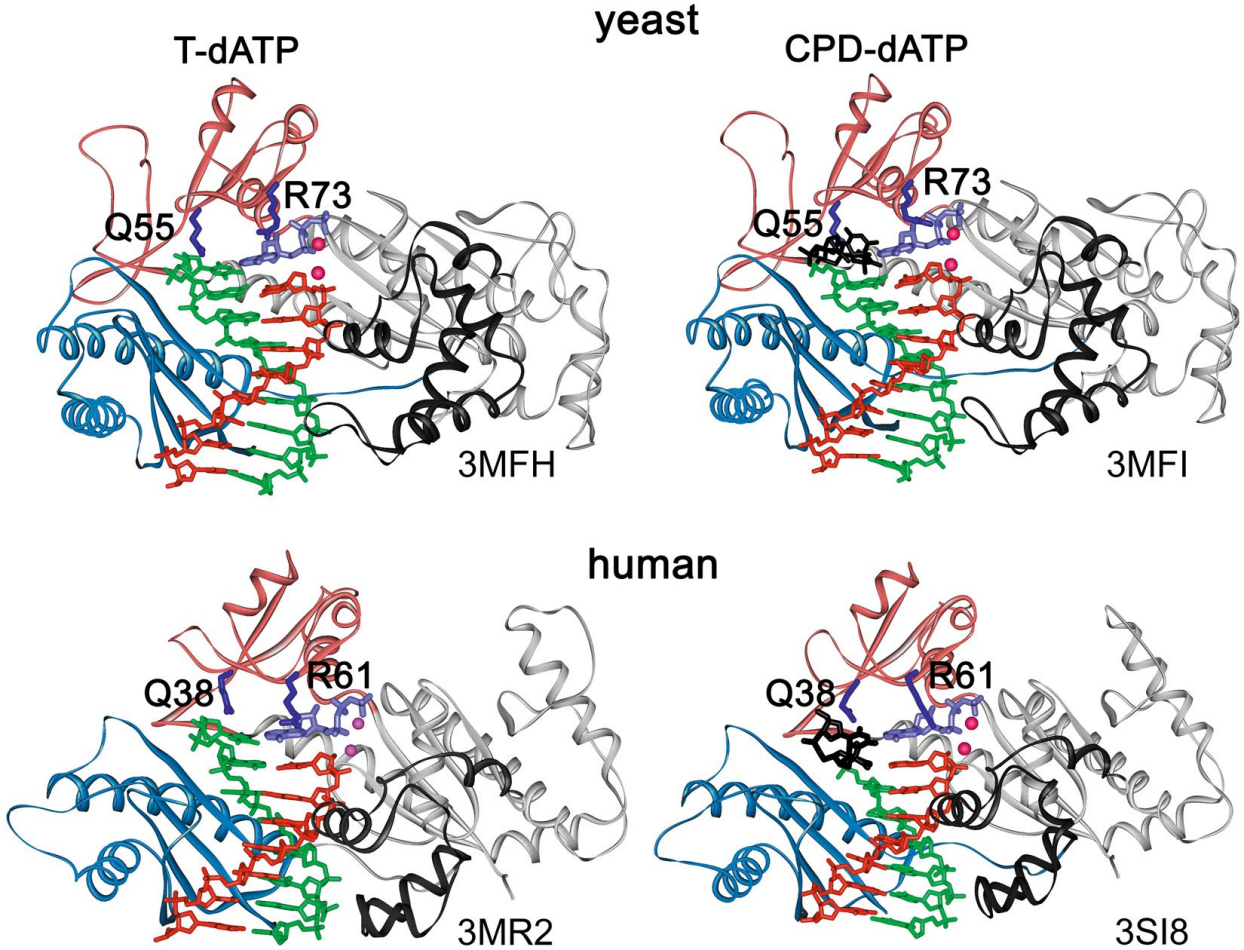
Structures of yeast (top^29^) and human (bottom^27^) Pol η in complex with DNA template (green) with undamaged T-T or T-T dimer (black), DNA primer (red) and incoming dATP (violet). The fingers domain is light carmine; the little finger domain is light blue; the thumb domain is black; the palm domain is light gray. *S. cerevisiae* Pol η Gln55 and Arg73 residues analyzed in this study and homologous Gln38 and Arg61 residues in human Pol η are colored in dark-blue.

Several crystal structures of the catalytic core of human and yeast Pol η in complex with undamaged DNA^6,26–31^, CPD^27,29^, cisplatin adducts^5–7^, 8-oxo-G^11,30,32^, AP-site^14^ and O^6^-me-G^18^ were solved. However, no structure is available for Pol η in a complex with TG-containing templates. Crystal structures revealed a right-hand-shaped architecture of Pol η that is similar to other Y-family DNA polymerases and consists of the palm, fingers, thumb and the little finger domains (Fig. 1). A distinctive feature of Pol η in comparison with other DNA polymerases, including related Y-family polymerases Pol ι and Pol κ, is a large and solvent-exposed active site which can accommodate two bases of the template strand or bulky CPD and cisplatin intrastrand crosslinks^5,6,27,29^. The loose fit of substrates in the enlarged active site can contribute to the low fidelity of Pol η^26^.

Active site residues Gln55 and Arg73 in the fingers domain of *S. cerevisiae* Pol η and the corresponding residues Gln38 and Arg61 in the human enzyme are uniquely conserved in Pol η and are involved in interactions with the template and the incoming nucleotides during catalysis (Fig. 1). According to the crystal structures, these residues maintain the stable configuration of T-T dimers during CPD bypass^27,29^. Moreover, these two residues in human Pol η also play a key role in the misincorporation of dGTP opposite template T by stabilizing the T-dGTP wobble base pair and may contribute to the mutagenesis at A/T base pairs in the WA motif during maturation of Ig genes^26^.

Biochemical studies supported a role for residues Gln38 and Arg61 in DNA replication by human Pol η. Mutations of these residues reduced the catalytic efficiency of human Pol η^27,30^. Substitution Q38A increased polymerase stalling after the CPD and substitution R61A reduced dGTP misinsertion opposite T^27^. Mutations of both residues also affected the spectrum of dNTP incorporation opposite 8-oxo-G and CPD^30,33^. However, experimental data on the roles of these residues in TLS opposite AP-sites and TG by human Pol η remain scarce.

In yeast Pol η, the single amino acid substitution Q55A displayed some loss in the DNA polymerase activity on undamaged DNA and opposite CPD^29^. However, substitution R73A had no effect on the activity on undamaged or T-T dimer-containing DNA^29^. The role of active site residues in TLS opposite other lesions (including 8-oxo-G, AP-site and TG) as well as in error-prone DNA synthesis by *S. cerevisiae* Pol η was not biochemically established.

To better understand the mechanism of DNA replication by Pol η and to further investigate the roles of *S. cerevisiae* Pol η Gln55 and Arg73 residues in TLS, we analyzed the effects of their alanine substitutions on the efficiency and fidelity of DNA synthesis on undamaged DNA as well as opposite CPD, AP-site, TG and 8-oxo-G lesions. We showed that both substitutions decreased the catalytic activity of full-length yeast Pol η, affected the spectrum of dNTP incorporation and significantly reduced the efficiency of DNA damage bypass by Pol η. We for the first time biochemically demonstrated the role of Gln55 and Arg73 in replication past AP-sites and TG by Pol η. We discuss the data in the light of the crystal structure of Pol η.

## Results

### Amino acid substitutions Q55A and R73A reduce the catalytic activity of *S. cerevisiae* Pol η

**Figure 2:**
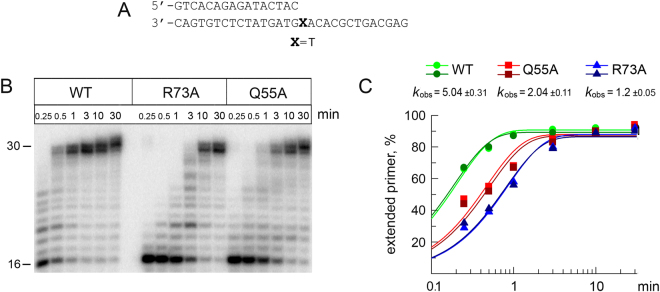
Primer extension by wild-type (WT), Q55A and R73A Pol η variants. (**A**) The structure of the DNA substrate used for the analysis. (**B**) Primer extension in the presence of four dNTPs. The reaction time was varied from 15 sec to 30 min. (**С**) Diagram showing the percentage of extended primer and calculated rate constants (*k*_obs_, min^−1^) with standard deviations. Two independent repeats of enzyme kinetics for each Pol η protein are shown.

To reveal the effects of the Q55A and R73A substitutions (Fig. 1) on the catalytic activity of Pol η we analyzed primer extension on a DNA template containing a T nucleotide downstream of the primer 3′-terminus (+1 position) (Fig. 2A). Reactions were terminated at different times after the addition of dNTPs. The time course of primer extension reactions showed that both substitutions moderately decreased the DNA polymerase activity of Pol η (Figs 1C and 2B), confirming the important roles of residues Gln55 and Arg73 in catalysis (see Introduction). The G55A and R73A substitutions reduced the catalytic rates of Pol η about 2.5- and 4-fold, correspondingly (Fig. 2C).

### Substitutions Q55A and R73A increase the fidelity of *S. cerevisiae* Pol η on undamaged DNA

**Figure 3:**
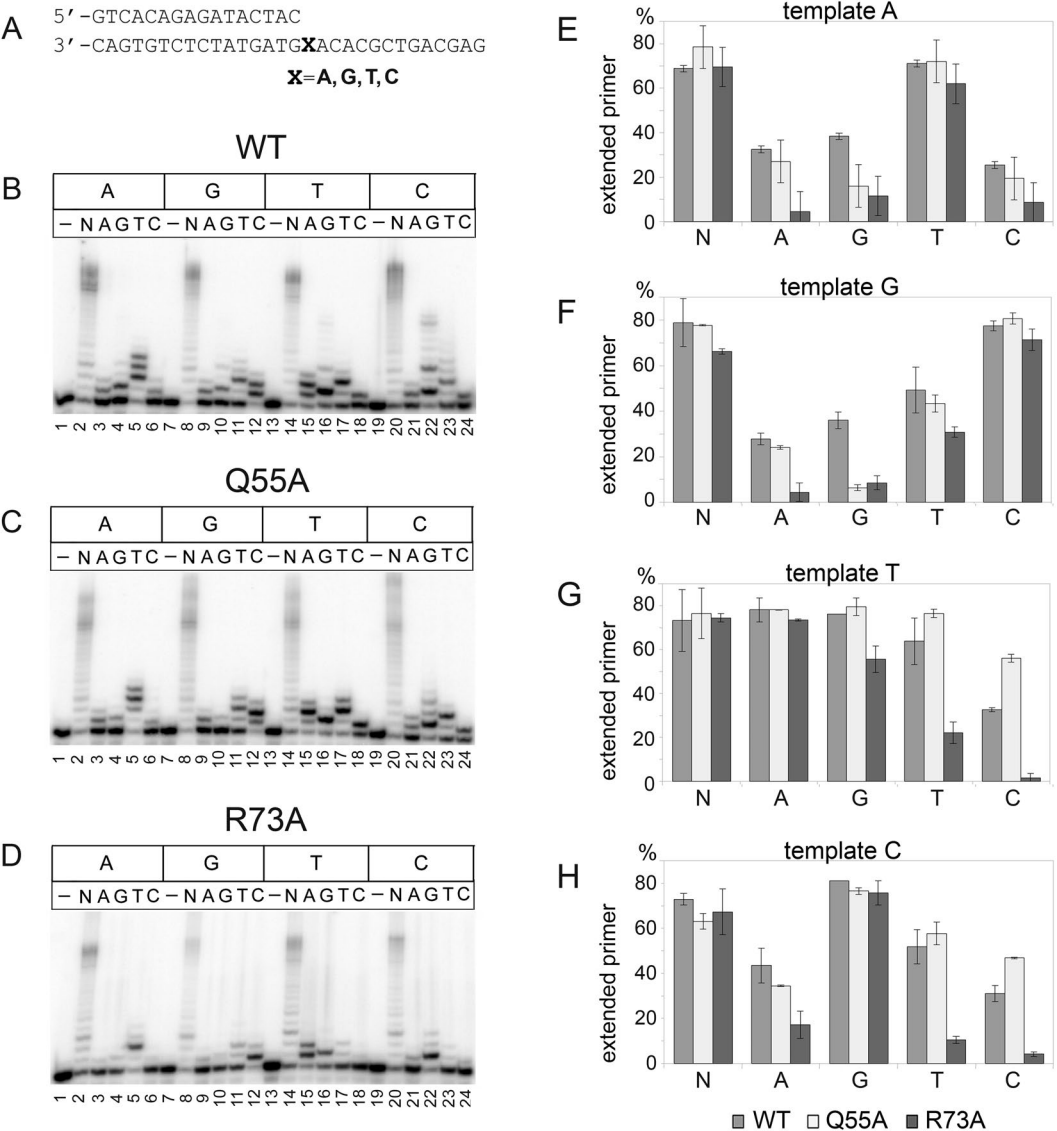
Primer extension by wild-type (WT), Q55A and R73A Pol η variants opposite undamaged templates A, G, T and C. (**A**) The structure of the DNA substrates used for the analysis. (**B**,**C**) and (**D**) Primer extension in the presence of four dNTPs (N), dATP (A), dGTP (G), dTTP (T) and dCTP (C). The reaction time was 5 min for wild-type Pol η and 20 min for Q55A and R73A Pol η variants. (**E**–**G**) and (**H**) Diagrams showing the percents of extended primers. Wild-type Pol η is gray; Q55A variant is light-gray; R73A variant is dark-gray. We note that the efficient incorporation of 4 dTTP opposite AACA (Fig. 3B, lane 5) can be explained by the efficient incorporation of dTTP opposite template C by Pol eta (Fig. 3B, lane 23) and long incubation time.

To analyze the fidelity of nucleotide incorporation by Pol η, we performed primer extension reactions with each individual dNTP on DNA templates containing either A, G, T, or C nucleotides at position +1 (Fig. 3A). To ensure equal DNA polymerase activities of wild-type Pol η and its mutant variants with amino acid substitutions, the reaction incubation time was extended for the mutant enzymes.

Both mutant Pol η variants revealed significant changes in the specificity of dNTP incorporation (Fig. 3B–H). The amino acid substitution R73A dramatically increased the Pol η fidelity. While the wild-type enzyme could incorporate all four individual dNTPs opposite all template nucleotides, the R73A polymerase incorporated almost exclusively correct nucleotides on templates A, G and C (Fig. 3D–F,H). Opposite template T the substitution R73A notably reduced misincorporation of pyrimidine dNTPs (Fig. 3D, lanes 17 and 18, Fig. 3G) and decreased misincorporation of dGTP (dGTP:T *p*-value = 0.04) (Fig. 3D, lane 16, Fig. 3G). This residue therefore likely plays a key and specific role in error-prone DNA replication by Pol η.

Substitution Q55A also increased the fidelity of Pol η opposite templating purines. In particular, the Q55A mutant could not efficiently incorporate dGTP opposite A and G (Fig. 3C, lanes 4 and 10, Fig. 3E,F). However this substitution increased misincorporation of dCTP opposite template pyrimidines (Fig. 3C, lanes 18 and 24, Fig. 3G,H).

### Substitutions Q55A and R73A affect DNA translesion synthesis opposite AP-site, TG and CPD by *S. cerevisiae* Pol η

**Figure 4:**
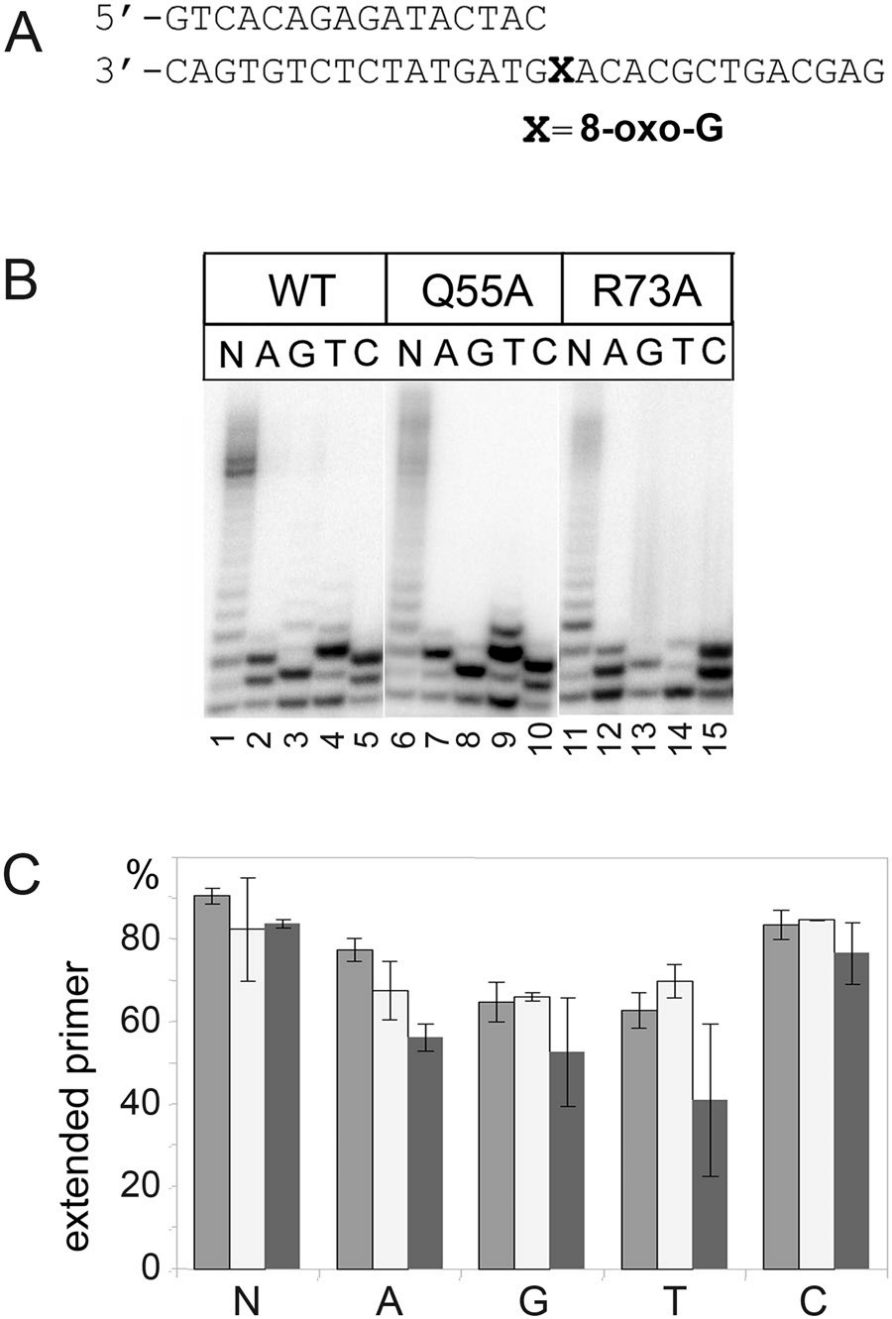
Primer extension by wild-type (WT), Q55A and R73A Pol η variants opposite 8-oxo-G. (**A**) The structure of the DNA substrate. (**B**) Primer extension in the presence of four dNTPs (N), dATP (A), dGTP (G), dTTP (T) and dCTP (C). The reaction time was 5 min for wild-type Pol η and 20 min for Q55A and R73A Pol η variants. (**C**) Diagram showing the percent of extended primers. Wild-type Pol η is gray; Q55A variant is light-gray; R73A variant is dark-gray.

We then analyzed the TLS activity of the Q55A and R73A mutants opposite different DNA lesions: AP-site, 8-oxo-G, CPD and TG. In accordance with published data, wild-type yeast Pol η efficiently replicated DNA past the 8-oxo-G lesion and preferably incorporated dCTP with a slight preference over other nucleotides (Figs 3C and 4B). Substitutions Q55A and R73A had almost no effect on the efficiency of 8-oxo-G bypass when all dNTPs were added to the reaction (Fig. 4B, lanes 6 and 10 and Fig. 4C). Substitution R73A slightly decreased incorporation of incorrect substrates opposite 8-oxo-G (*p*-value for dATP:8-oxo-G = 0.015; *p*-value for dTTP:8-oxo-G = 0.068) (Fig. 4B, lanes 12–14 and Fig. 4C) while substitution Q55A did not change the specificity of dNTPs incorporation opposite the lesion (Fig. 4B, lanes 7–10 and Fig. 4C).

**Figure 5:**
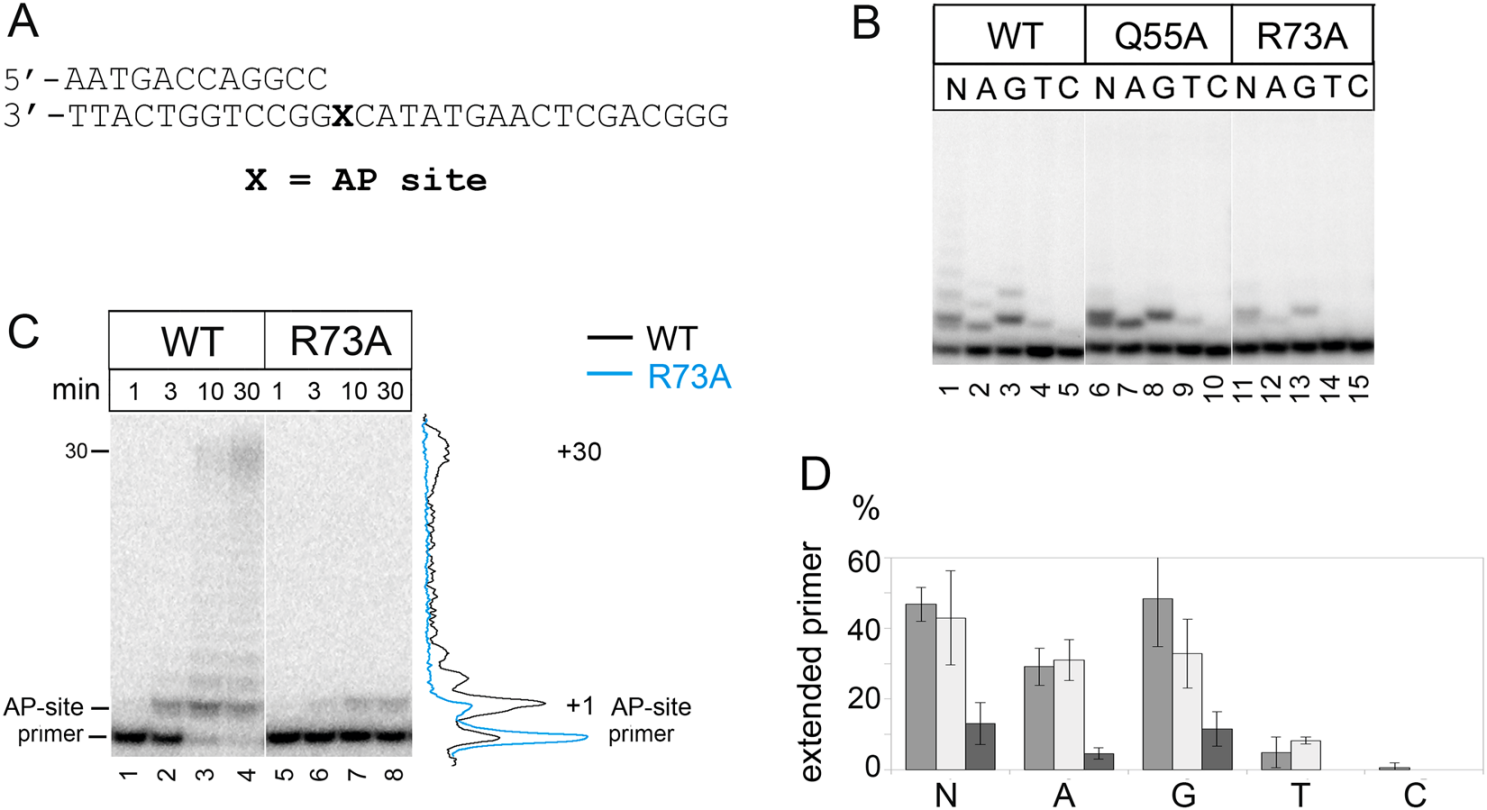
Primer extension by wild-type (WT), Q55A and R73A Pol η variants opposite AP-site. (**A**) The structure of the DNA substrate. (**B**) Primer extension in the presence of four dNTPs (N), dATP (A), dGTP (G), dTTP (T) and dCTP (C). The reaction time was 5 min for wild-type Pol η and 20 min for Q55A and R73A Pol η variants. (**C**) Primer extension in the presence of four dNTPs. The reaction time was varied from 1 to 30 min. (**D**) Diagram showing the percent of extended primers (for Fig. 5B). Wild-type Pol η is gray; Q55A variant is light-gray; R73A variant is dark-gray.

In agreement with published data, wild-type yeast Pol η preferably incorporated dATP and dGTP opposite AP-site with a preference over dCTP and dTTP (Fig. 5B, lanes 2–5, and Fig. 5D). The efficiency of dGTP incorporation (expressed as V_max_*/K*_M_) was 2.4-fold higher compared to dATP (Table 1). However, Pol η inefficiently extended the primer beyond AP-site (Fig. 5B, lane 1, and Fig. 5C, lanes 1–4). Substitution Q55A had no effect on the AP-site bypass (Fig. 5B, lanes 6–10, and Fig. 5D). In contrast, the R73A mutation significantly reduced the efficiency of incorporation of all dNTPs opposite the AP-site (Fig. 5B, lanes 12–15 and Table 1) and fully blocked replication after the lesion (Fig. 5C, lanes 5–8). In particular, the R73A mutation reduced incorporation of dATP and dGTP opposite the AP-site 11- and 7-fold, respectively (Table 1). These data suggest that residue Arg73 plays an important role in the TLS activity of Pol η on DNA with AP-site.

**Table 1:** Steady-state kinetic parameters for dNTP incorporation opposite AP-site by WT Pol η and R73A Pol η variant.

enzyme	dNTP	K_M_, μM	V_max_, % per min	V_max_/K_M_	V_max_*/K*_M_ (relative to WT)
WT	dATP	129.75 ± 3	16.58 ± 0.07	1.28 × 10^−1^	1
	dGTP	74.05 ± 0.08	21.73 ± 0.27	2.93 × 10^−1^	1
R73A	dATP	439.28 ± 13.65	5.15 ± 0.34	1.17 × 10^−2^	0.09
	dGTP	119.34 ± 3	5.1 ± 0.05	4.27 × 10^−2^	0.15

**Figure 6:**
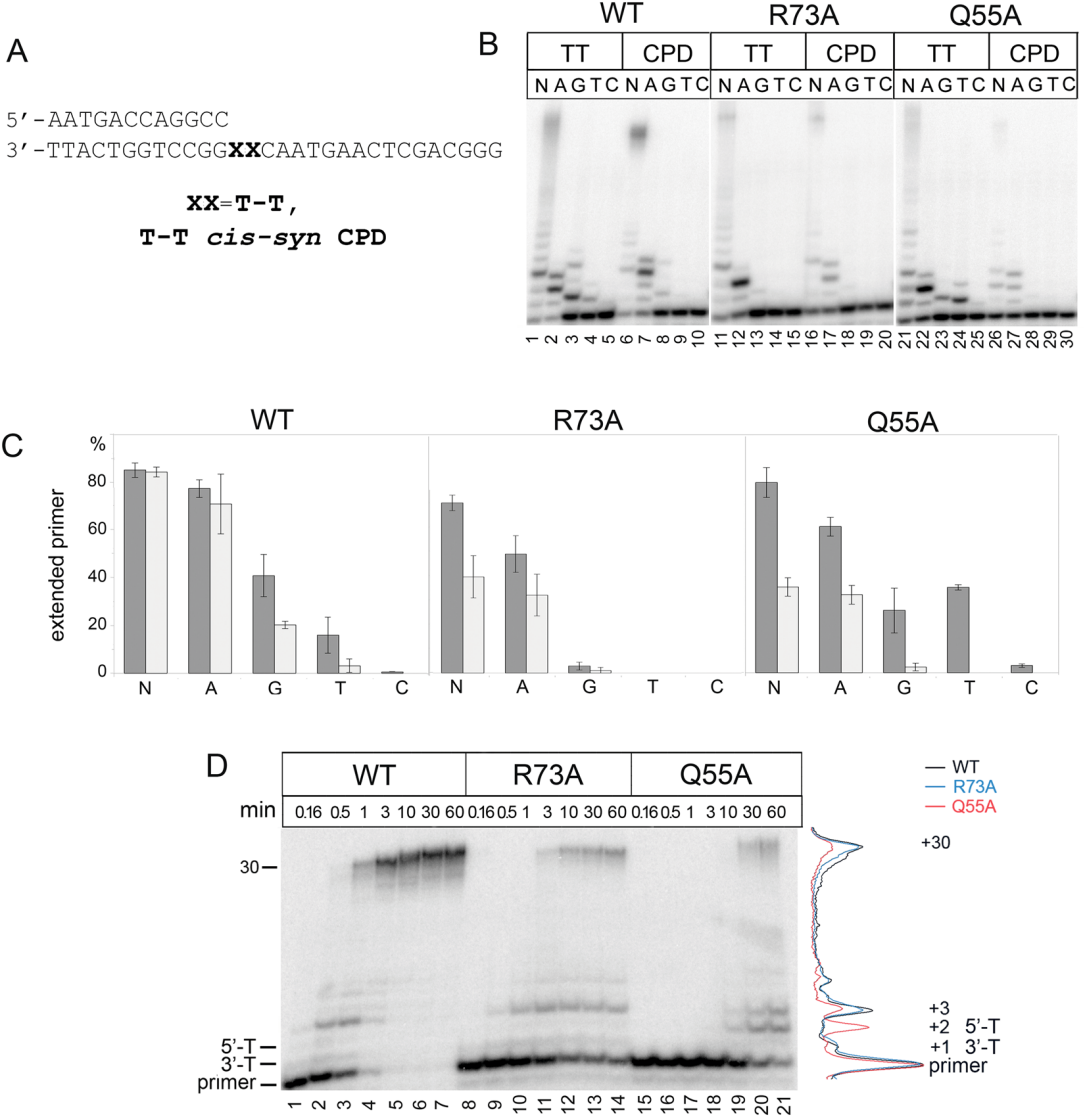
Primer extension by wild-type (WT), Q55A and R73A Pol η variants opposite CPD. (**A**) The structure of the DNA substrates. (**B**) Primer extension in the presence of four dNTPs (N), dATP (A), dGTP (G), dTTP (T) and dCTP (C). The reaction time was 5 min for wild-type Pol η and 20 min for Q55A and R73A Pol η variants. (**C**) Diagram showing the percent of extended primers. Undamaged T-T is gray and CPD is light-gray. (**D**) Primer extension in the presence of four dNTPs. The reaction time was varied from 10 sec to 60 min.

We next analyzed the TLS activity of yeast Pol η opposite CPD. As expected, wild-type yeast Pol η efficiently and accurately bypassed the CPD lesion (Fig. 6B–D and Table 2). Both R73A and Q55A substitutions slightly increased the fidelity of dNTPs incorporation opposite CPD; in particular, no misincorporation of dGTP was observed (Fig. 6B, lanes 18 and 28, and Fig. 6C,D). Both substitutions also reduced the efficiency (V_max_*/K*_M_) of correct dATP incorporation opposite CPD, with a stronger effect for the Q55A Pol η variant. In particular, the Q55A mutation increased *K*_M_ for dATP incorporation opposite CPD ~ 22-fold and decreased the V_max_*/K*_M_ value ~140-fold in comparison with the wild-type enzyme (Table 2).

**Table 2:** Steady-state kinetic parameters for dATP incorporation opposite CPD by WT Pol η and Q55A and R73A Pol η variants.

enzyme	template	K_m_, μM	V_max_, % per min	V_max_/ K_m_	V_max_*/K*_M_ (relative to undamaged template)	V_max_*/K*_M_ (relative to WT)
WT	CPD	5.91 ± 1.06	57.41 ± 6.81	9.71	0.27	1
	TT	1.83 ± 0.9	56.56 ± 4.25	30.91	1	1
Q55A	CPD	130.74 ± 14.63	8,94 ± 0,41	6.84 × 10^−2^	0.09	0.007
	TT	25.82 ± 3.85	18.8 ± 0.32	7.28 × 10^−1^	1	0.02
R73A	CPD	9.38 ± 3.92	8.44 ± 0.89	9 × 10^−1^	0.03	0.09
	TT	3.75 ± 0.16	28.57 ± 0.29	7.62	1	0.25

The reduced TLS activity opposite CPD may be partially explained by the decrease in the catalytic activity of Pol η variants. The strong reduction of dATP incorporation was also observed opposite template with undamaged TT (Table 2). However, the Q55A variant also significantly reduced the efficiency of primer extension after CPD. The mutant Pol η stalling was observed at the +2 template position downstream of the primer, corresponding to the 5′-T of the T-T dimer (Fig. 6B, lanes 26 and 27, and Fig. 6D, lanes 19–21). The accumulation of DNA products at the +2 T position was not observed on the undamaged template (Fig. 6D, lanes 1–7) and was therefore specific for the CPD lesion. These data are in agreement with the key role of residue Gln55 in the efficient bypass of T-T dimers by Pol η.

**Figure 7:**
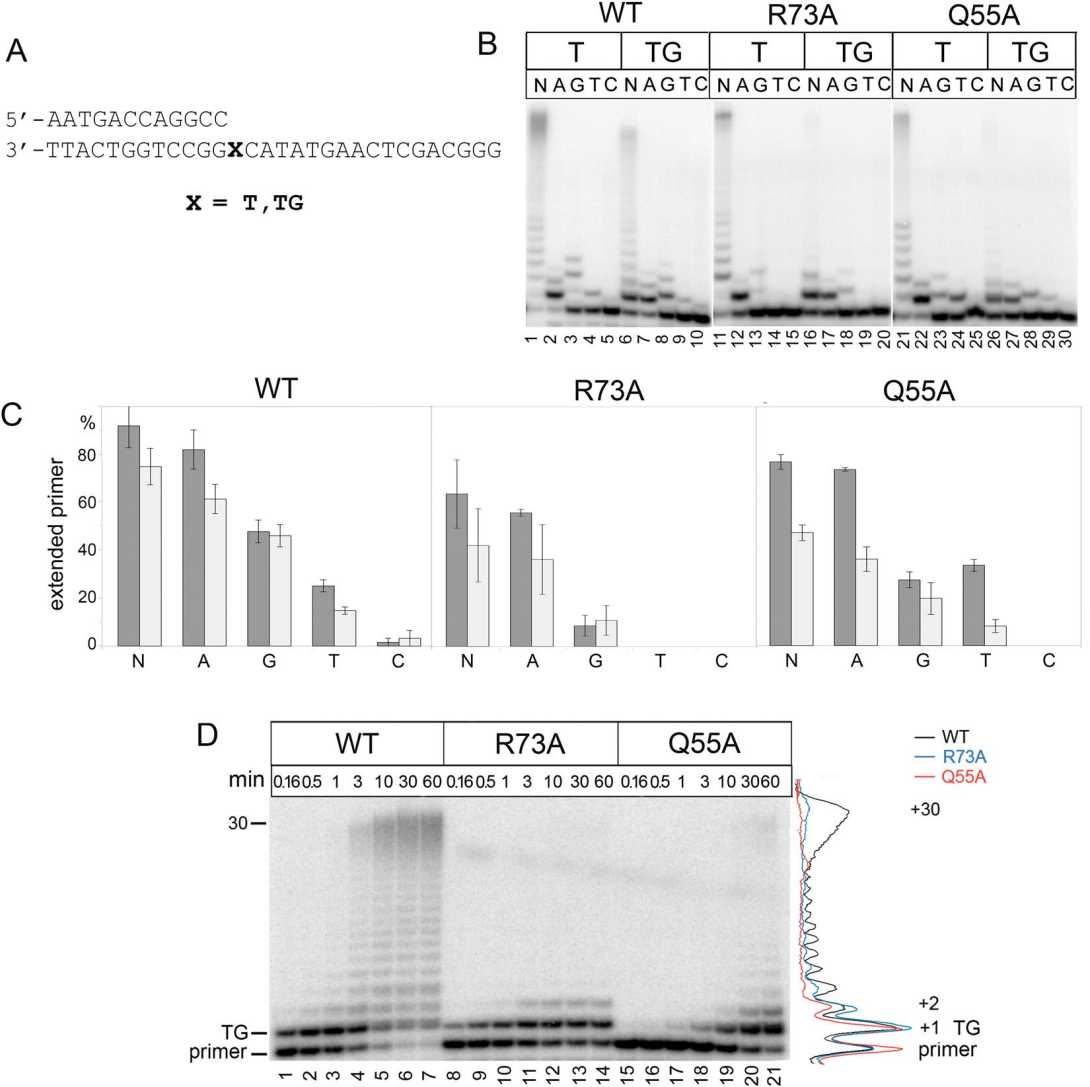
Primer extension by wild-type (WT), Q55A and R73A Pol η variants opposite TG. (**A**) The structure of the DNA substrates used for the analysis. (**B**) Primer extension in the presence of four dNTPs (N), dATP (A), dGTP (G), dTTP (T) and dCTP (C). The reaction time was 5 min for wild-type Pol η and 20 min for Q55A and R73A Pol η variants. (**C**) Diagram showing the percent of extended primers. Undamaged T is gray and TG is light-gray. (**D**) Primer extension in the presence of four dNTPs. The reaction time was varied from 10 sec to 60 min.

Finally, we investigated bypass of the TG lesion by yeast Pol η. We observed efficient and accurate nucleotide incorporation opposite TG by the wild-type Pol η (Fig. 7B, lanes 6–10 and Fig. 7C, and Table 3). Pol η incorporated complementary dATP opposite TG only ~ 2.5-fold less efficiently than opposite undamaged T (Table 3). Pol η was also able to replicate DNA beyond the lesion (Fig. 7D, lanes 1–7). In particular, >50% of the primer was extended after 10 min. For comparison, >50% of CPD and AP-site were bypassed by Pol η after 3 min and 30 min, respectively (Fig. 5C, lane 3, Fig. 6D, lane 3 and Fig. 7D, lane 4).

**Table 3:** Steady-state kinetic parameters for dNTP incorporation opposite TG by WT and Q55A and R73A Pol η variants.

enzyme	template	dNTP	K_M_, μM	V_max_, % per min	V_max_/K_M_	V_max_*/K*_M_ (relative to undamaged template)	V_max_*/K*_M_ (relative to WT)
WT	TG	dATP	3.79 ± 0.19	40.64 ± 2.16	10.7	0.4	1
		dTTP	201.1 ± 53.9	12.21 ± 1.68	6.1 × 10^−2^	—	1
	T	dATP	1.99 ± 0.27	53.74 ± 5.75	27	1	1
Q55A	TG	dATP	17.75 ± 4.01	8.11 ± 0.38	4.57 × 10^−1^	0.41	0.04
		dTTP	227.01 ± 54.31	2.93 ± 0.28	1.29 × 10^−2^	—	0.21
	T	dATP	13.95 ± 0.87	15.72 ± 0.78	1.13	1	0.04
R73A	TG	dATP	3.22 ± 0.65	9.4 ± 0.27	2.92	0.41	0.28
		dTTP	489.9 ± 188.9	1.71 ± 0.29	3.49 × 10^−3^	—	0.06
	T	dATP	4.15 ± 0.43	29.49 ± 1.44	7.11	1	0.26

Both Gln55 and Arg73 were found to be important for the efficient TG bypass by Pol η (Fig. 7). Pol η variants with the Q55A and R73A substitutions showed reduced efficiency of nucleotide incorporation opposite TG (Table 3). Reduction of dATP incorporation was also observed opposite undamaged T and was not specific to the TG lesion. However, Q55A and R73A mutant variants were unable to extend the primer beyond the damaged nucleotide (Fig. 7B, lanes 16, 17, 26, 27 and Fig. 7D, lanes 8–21).

## Discussion

### Roles of residues Gln55 and Arg73 in fidelity of DNA synthesis by *S. cerevisiae* Pol η

Both the Q55A and R73A substitutions moderately decreased the DNA polymerase activity of Pol η and altered the spectrum of dNTPs incorporation on undamaged DNA. In contrast with published data^29^, the R73A substitution had a stronger effect on the catalytic activity of yeast Pol η comparing to the Q55A substitution in our study.

While substitution Q55A reduced misincorporation of dGTP opposite template purines but increased misincorporation of dCTP opposite template pyrimidines, substitution R73A significantly decreased incorporation of non-complementary dNTPs opposite all templating nucleotides. Previously, it was shown that corresponding substitution (R61A) in human Pol η decreased the misincorporation of dGTP opposite template T^27^, increased the fidelity of DNA synthesis opposite templating purines and reduced misincorporation of dCTP opposite T^33^. Our data are in agreement with these observations and further extend the role of Arg73 in error-prone DNA replication opposite all templating nucleotides in the yeast enzyme.

According to the structural data, Gln55/Gln38 interacts with templating bases in the minor groove during catalysis (Fig. 1)^27,29,31^. While these contacts may play a role in the incorporation of dGTP opposite template purines by positioning the template base in a specific conformation, we propose that this residue may also directly participate in contacts with the incoming dGTP. Indeed, in one of published structures of human Pol η residue Gln38 makes a direct hydrogen bond with the N^2^ atom of the incoming dGTP (bound opposite a 1,N^6^-ethenoadenine template base). Therefore, the function of this residue may be similar to the function of the corresponding residue Gln59 in human Pol η, in which it also hydrogen-bonds with the incoming dGTP^34^ and its substitutions reduce dGTP misincorporation^35^.

According to the yeast and human Pol η-DNA structures, the Arg73/Arg61 residue is flexible. While in most structures Arg73/Arg61 interacts with the base and phosphates of the incoming dNTP, it can also adopt a rotamer conformation and interact with the next templating nucleotide at position + 2^27,29,31^. Such flexibility of Arg73 in contacts with DNA and incoming nucleotides might promote the incorporation of non-complementary nucleotides. Interestingly, decreased dGTP misincorporation opposite template T by the R73A mutant was observed on a DNA template with the “TA” context but was almost completely abolished in the case of the “TT” context (compare Fig. 3D, lane 16 and Fig. 3H with Fig. 6B, lane 13 and Fig. 6C). Thus, the DNA sequence context makes contribution to the error-prone activity of Pol η and modulates the specificity of dNTP misincorporation by the R73A mutant. It is likely that interactions of Arg73 with DNA could stabilize the template conformation and increase misincorporation of non-complementary dNTPs by the wild-type Pol η The loss of the contacts with + 2 nucleotide in the case of the R73A mutant might explain the increased fidelity of this mutant, depending on the sequence context.

### Roles of residues Gln55 and Arg73 in the TLS-activity of *S. cerevisiae* Pol η

Similarly with published results^1,2,29^, we demonstrated that yeast Pol η has the highest accuracy and fidelity on DNA templates with *cis–syn* thymine dimers. It was shown that both Gln55 and Arg73 are important for replication across CPD but substitution of Gln55 had a stronger effect on TLS opposite the lesion. Substitution Q55A significantly reduced the efficiency of dATP incorporation opposite T-T CPD and abolished the extension of DNA synthesis beyond the lesion. The key role of these residues in CPD bypass is in agreement with previous structural and biochemical data for *S. cerevisiae* Pol η^29^ and human Pol η^27^. In particular, in published Pol η structures with CPD templates, residue Gln55 forms a hydrogen bond with O2 atom of the 3′-T of T-T dimer while the guanidinium group of Arg73 contacts the incoming dATP^29^.

The lowest Pol η TLS-activity among the tested DNA lesions was observed opposite the AP-site. In agreement with previous data^16^, yeast Pol η preferably incorporated purine nucleotides with preference over pyrimidine nucleotides, and the extension beyond the AP-site was inefficient. In contrast with CPD, Arg73 was shown to play a key role in the AP-site bypass. Substitution R73A reduced the efficiency of dNTP insertion opposite the AP-site without affecting the spectrum of nucleotide incorporation and fully blocked further DNA extension beyond the AP-site. These data with the yeast enzyme are in agreement with the recently published structures of human Pol η with tetrahydrofuran analog of AP-site and incoming non-hydrolyzable analogs of dATP or dGTP (dAMPNPP and dGMPNPP)^14^. In the complex with dGMPNPP, the side chain of Arg61 in human Pol η interacts with the Hoogsteen edge (O6 and N7) of guanine base. In the dAMPNPP complex, Arg61 adopts two alternative conformations and either participates in stacking with the base or interacts with the α- and β-phosphates of the incoming nucleotide^14^. These data explain the “purine” rule in dNTPs incorporation opposite the AP-site and suggest a key evolutionary conserved role of residue Arg73/Arg61 in the stabilization of incoming dATP and dGTP nucleotides in the active site of Pol η.

In agreement with published data^8^, yeast Pol η efficiently replicated DNA past the 8-oxo-G lesion and incorporated all four nucleotides with a slight preference for dCTP and dATP over dTTP and dGTP. However, we showed that neither Q55A nor R73A substitution had significant effect on the spectrum of dNTPs incorporation and the efficiency of 8-oxo-G lesion bypass by the yeast enzyme. It was suggested that the yeast Pol η active site is well adapted to accommodate an 8-oxo-G lesion in the *anti* conformation for Watson-Crick base pairing with incoming dCTP^32^. Multiple contacts between active side residues and DNA template were observed for both undamaged G and 8-oxo-G (hydrogen bonds between G/8-oxo-G and Gln55, Trp56 and Asn400 as well as a hydrogen bond and van der Waals interactions between the nucleotide 5′ to the templating 8-oxo-G and Arg73 and Met74). Moreover, the 8-oxo-G lesion is specifically stabilized in the *anti* conformation by a direct and a water-mediated hydrogen bond between the O8 of 8-oxo-G and residues Asn398 and Asn400 of the Pol η active site^32^. It is therefore possible that contacts of residues Gln55 and Arg73 with the DNA template are not essential for the stabilization of 8-oxo-G and for the TLS activity of yeast Pol η across the lesion. Interestingly, it was shown that in the complex of human Pol η with 8-oxo-G, polarized water molecules mimic and partially compensate for the missing wild-type side chains when Arg61 and Gln38 residues were replaced by alanines^30^. It is possible that similar effects might explain the lack of *in vitro* defects in yeast Pol η Q55A and R73A mutants during DNA synthesis opposite 8-oxo-G.

Finally, we reported that yeast Pol η carries out efficient and accurate replication across another common oxidative DNA lesion, thymine glycol. Previous studies demonstrated that human Pol η catalyzes insertion of correct dATP opposite TG as efficient as opposite undamaged T, while further primer extension is somewhat inhibited^9^. Similarly, the efficient and accurate bypass of TG was observed *in vitro* for Pol η from thermophilic worm *Alvinella pompejana*^36^. Therefore, the ability of Pol η to replicate across TG is likely conserved among eukaryotic organisms. Furthermore, substitutions of residues Gln55 and Arg73 in yeast Pol η impaired DNA extension beyond the lesion.

**Figure 8:**
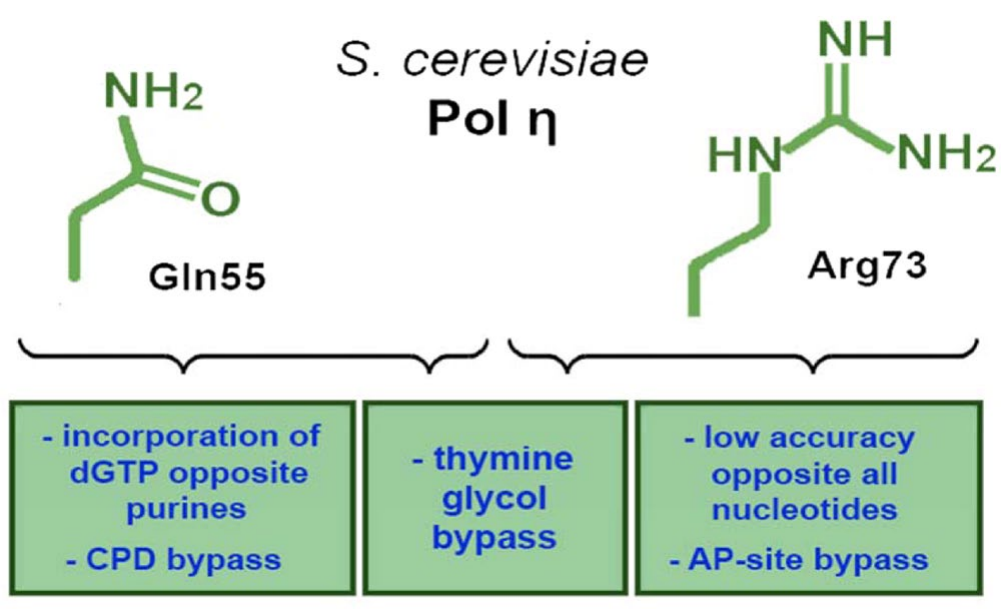
The roles of Gln55 and Arg73 residues in the TLS activity and error-prone DNA synthesis by yeast Pol η.

The functions of residues Gln55 and Arg73 in the TLS activity and error-prone DNA synthesis by yeast Pol η are summarized in Fig. 8. Our results reveal a key role of these residues in DNA synthesis opposite various (but not all) types of DNA lesions as well as in error-prone behavior of Pol η on undamaged DNA. Substitutions of these residues reduced the TLS activity of Pol η opposite many DNA lesions but increased the fidelity of DNA synthesis. Our observations are consistent with the studies of the role of homologous residues in human Pol η. It is therefore likely that these conserved residues were selected during evolution to maximize the efficiency and accuracy of translesion DNA synthesis. Previously, we showed that single amino acid substitutions in the fingers domain dramatically increased the fidelity of another Y-family TLS polymerase – Pol ι^35^. These findings highlight the evolutionary importance of the TLS function of Y-family DNA polymerases at the cost of accuracy of DNA replication.

## Methods

### Purification of wild-type Pol η and its mutant variants

Mutant forms of the *RAD30* gene encoding for yeast Pol η with amino acid substitutions Q55A and R73A were constructed using the Quick Change mutagenesis kit (Agilent) in the plasmid pBL810^37^. GST-tagged full-length wild type *S. cerevisiae* Pol η and its mutant variants were produced in yeast strain BJ 2168 and purified as described for yeast Rev1 and Pol ζ^38,39^. All proteins were >95% pure.

### DNA oligonucleotide substrates

DNA templates with T-T dimer, tetrahydrofuran (a stable analog of AP-site), TG and 8-oxo-G were purchased from The Midland Certified Reagent Company (Midland, USA) and Trilink Biotechnologies, Inc. (San Diego, USA). Unmodified primers and corresponding undamaged template DNA oligonucleotides were synthesized by Syntol (Moscow, Russia). To obtain DNA substrates the primers were 5′-labeled with [γ-^32^P]-ATP using T4 polynucleotide kinase, mixed with corresponding unlabeled template oligonucleotides at a molar ratio of 1:1.1, heated to 73 °C and slowly cooled down to 20 °C.

### Primer extension reactions

Standard primer extension reactions were carried out in 20 μl of reaction buffer containing 40 mM HEPES (pH 7.4), 2.5% glycerol, 0.1 mg/ml bovine serum albumin, 10 mM MgCl_2_, 20 nM DNA substrate, 100 μM dNTPs and 10 nM of Pol η. Reactions were started by adding of dNTPs and incubated at 37 °C. Reactions with wild-type Pol η were incubated for 5 min and reactions with mutant Pol η variants were incubated for 20 min. The reactions were terminated by the addition of an equal volume of loading buffer (95% formamide, 10 mM EDTA, 0.1% bromophenol blue and 0.1% xylene cyanol). DNA products were resolved on 23% polyacrylamide gels containing 8 M urea, followed by phosphorimaging. The percentage of extended primer was calculated as the ratio between the signal of all extended products and the total lane signal for each reaction. Experiments were repeated three times; the standard deviations were calculated and were shown as error bars on graphs.

### Calculation of DNA polymerization rate constants

To measure the rate of DNA synthesis, primer extension reactions were stopped after various time intervals (15 sec to 30 min, as specified in figure legends). To calculate the *k*_obs_ values of the reaction, the kinetics of the primer extension were fit to a single exponential equation: A = A_max_ × [1 − exp(−*k*_obs_ × t)] using a nonlinear regression, where A is the efficiency of primer extension (the observed % of extended primer), A_max_ is the maximal primer extension, *k*_obs_ is the observed first-order rate constant for primer extension and t is the reaction time. Calculations were made using GraFit software (Erithacus Software, UK). Experiments were repeated two times.

### Steady-state kinetics analysis of individual dNTP incorporation

To quantify the incorporation of individual dNTPs we varied each dNTP concentration from 0.003 to 1000 μM in reactions. Reaction mixtures were incubated for different time intervals (from 30 sec to 20 min). The data were fit to the Michaelis-Menten equation V = V_MAX_ × [dNTP])/(K_M_ + [dNTP]) using a nonlinear regression in GraFit software, where V and V_MAX_ is the observed and the maximum rates of the reaction (in % of utilized primer per 1 min), respectively, and K_M_ is the apparent Michaelis constant. Calculated apparent K_M_ and V_MAX_ parameters were used to determine the catalytic efficiency (V_MAX_/K_M_) and the fidelity of dNTP incorporation (V_MAX_/K_M_ for incorrect dNTP divided by V_MAX_/K_M_ for the correct substrate). Experiments were repeated three times. All calculations and comparisons between DNA lesions and corresponding undamaged nucleotides were made in the same DNA sequence context.

### Data availability

The data that support the findings of this study are included in the Supplementary Information file or are available from the corresponding author on request. Expression constructs for yeast Pol η with amino acid substitutions Q55A and R73A are available from the corresponding author on request.

## Electronic supplementary material

Supplementary information
