# Supplementary material for: The active site residues Gln55 and Arg73 play a key role in DNA damage bypass by S. cerevisiae Pol η
Source: Sci Rep. 2018 Jul 9;8:10314. doi: 10.1038/s41598-018-28664-8 (PMC6037775; doi:10.1038/s41598-018-28664-8)
Supplement: Supplementary file 1 — Supplementary information [file 41598_2018_28664_MOESM1_ESM.pdf]

The active site residues Gln55 and Arg73 play a key role in DNA damage bypass by *S. cerevisiae* Pol η

Elizaveta O. Boldinova, Artem Ignatov, Andrey Kulbachinskiy and Alena V. Makarova*

* corresponding author
Institute of Molecular Genetics, Russian Academy of Sciences, Kurchatov sq. 2, 123182 Moscow, Russia; tel: (499) 196 0015; E mail: amakarova-img@yandex.ru

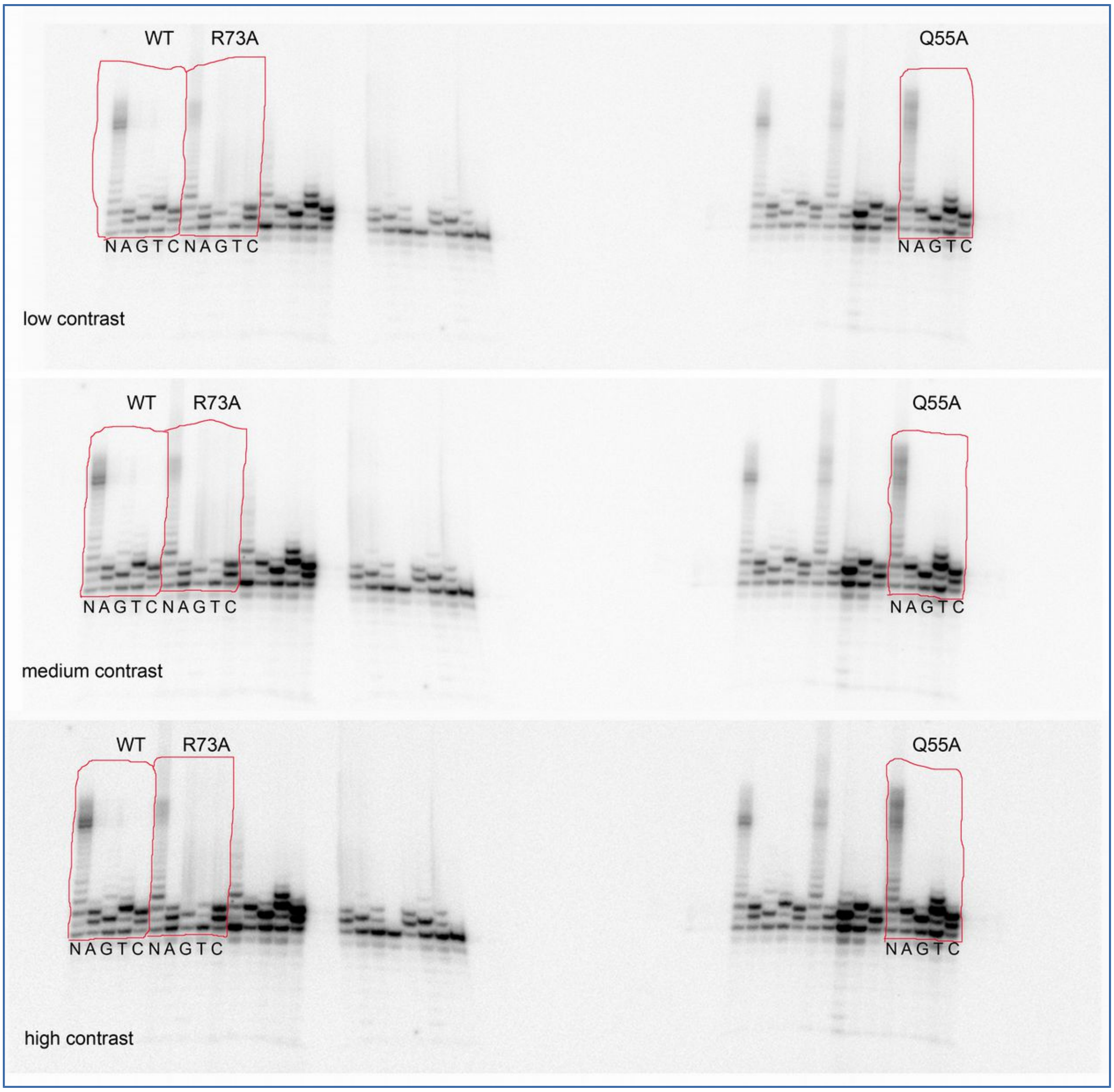

The original gel for Fig. 4B with different contrasts. Parts of the gel used in Fig 4B are highlighted in red pencil.

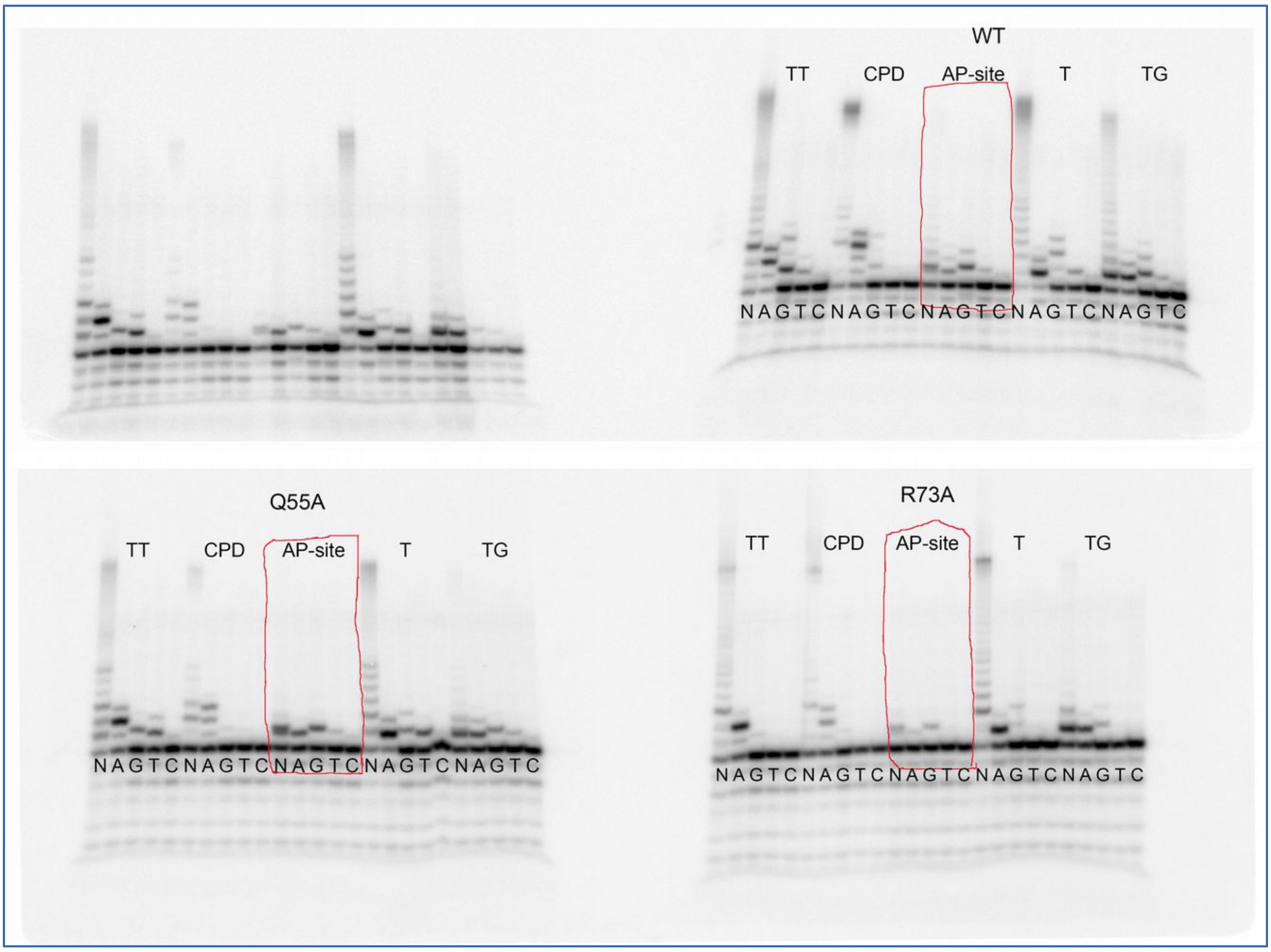

The original gels for Fig. 5B. Parts of the gels used in Fig 5B are highlighted in red pencil.

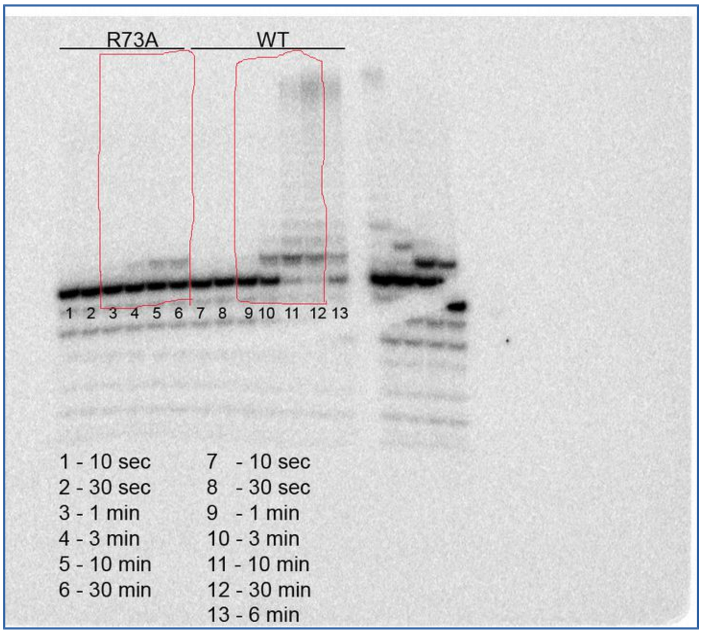

The original gel for Fig. 5C. Parts of the gel used in Fig 5C are highlighted in red pencil.

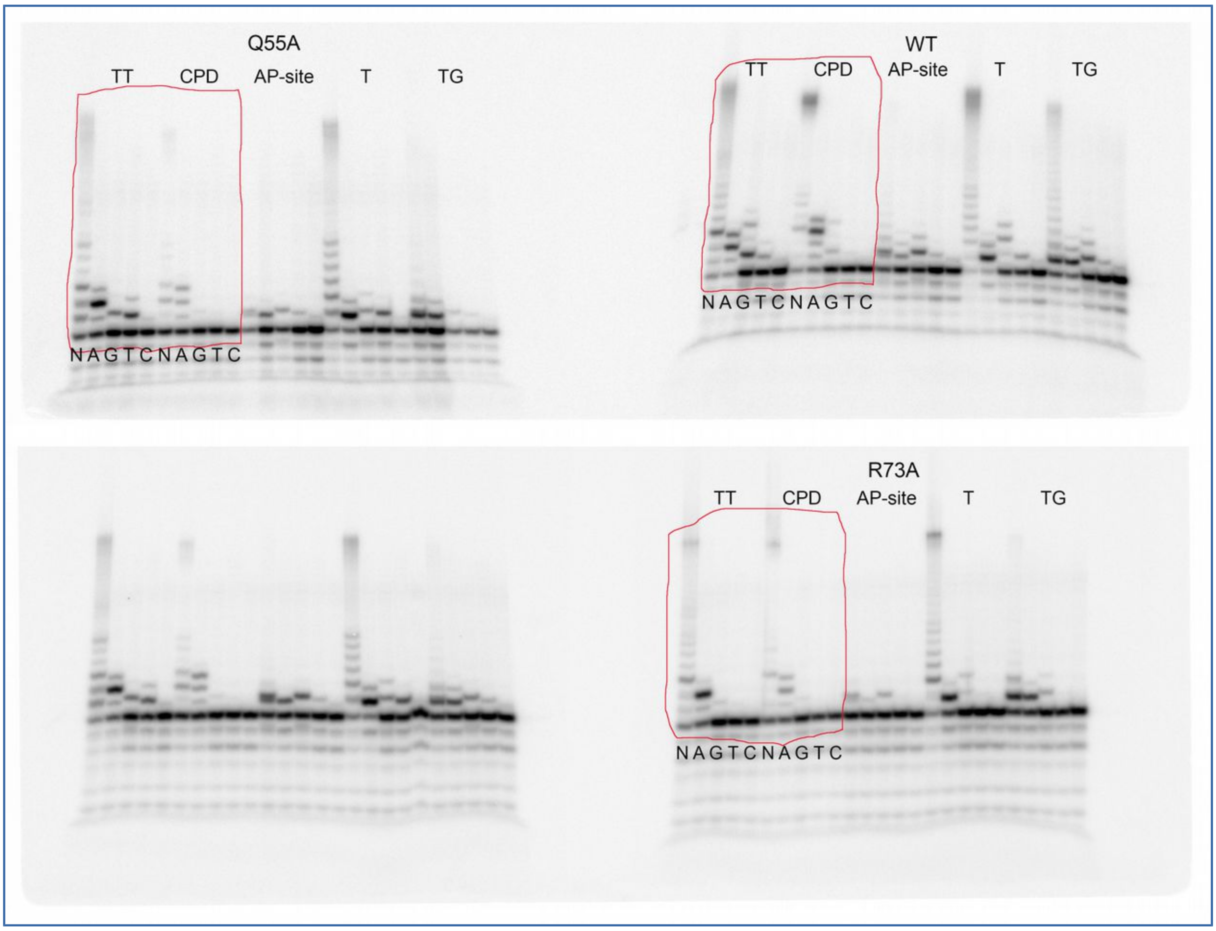

The original gels for Fig. 6B. Parts of the gels used in Fig 6B are highlighted in red pencil.

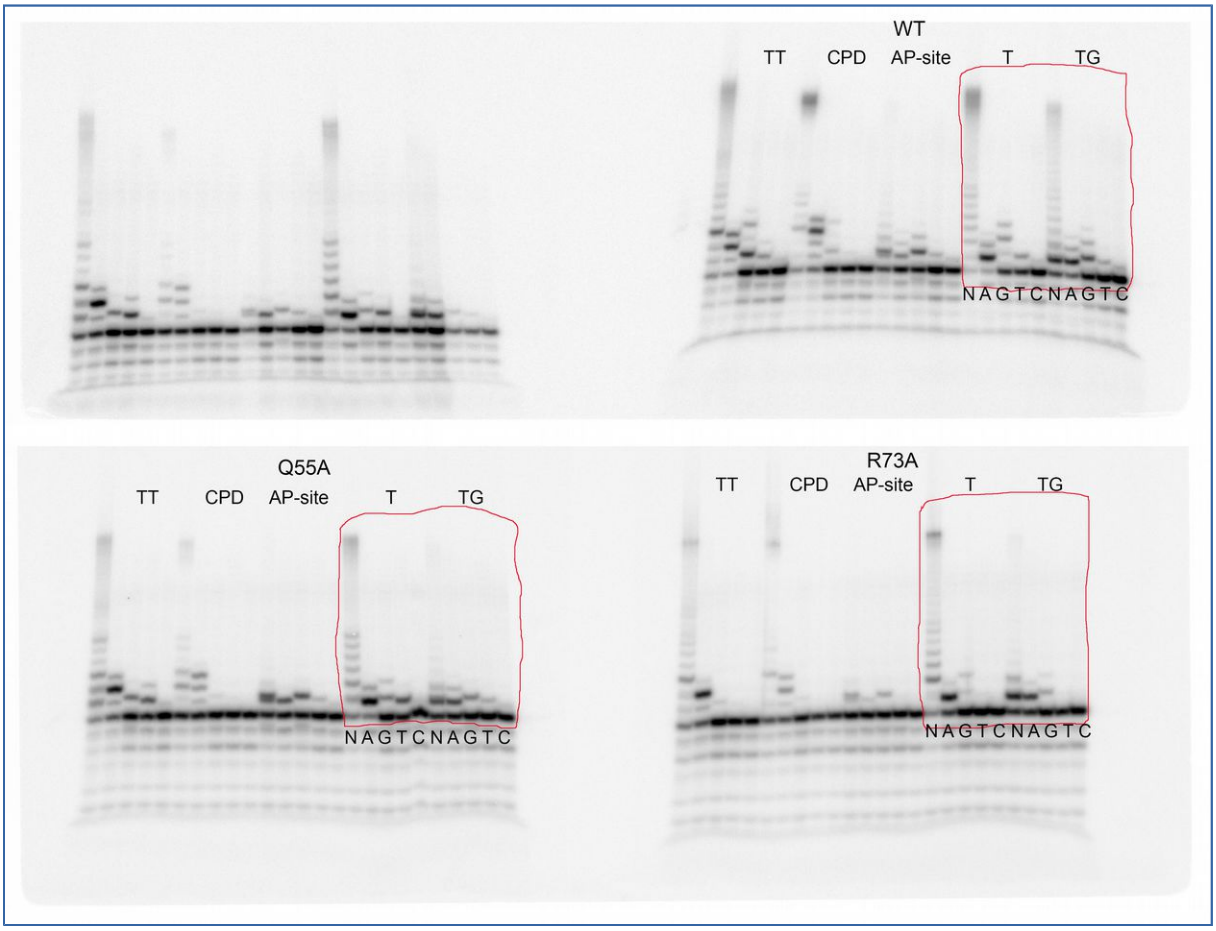

The original gels for Fig. 7B. Parts of the gels used in Fig 7B are highlighted in red pencil.
